# Inhibition of angiogenesis and tumor progression of MK-0429, an integrin αvβ_3_ antagonist, on oral squamous cell carcinoma

**DOI:** 10.1007/s00432-022-04100-3

**Published:** 2022-06-17

**Authors:** Takayuki Nakagawa, Kouji Ohta, Takako Naruse, Miyuki Sakuma, Syohei Fukada, Nao Yamakado, Misaki Akagi, Kazuki Sasaki, Chieko Niwata, Shigehiro Ono, Tomonao Aikawa

**Affiliations:** 1 Department of Oral and Maxillofacial Surgery, Program of Dentistry, Graduate School of Biomedical and Health Sciences, Hiroshima University, Kasumi 1-2-3, Minami-Ward, Hiroshima, 734-8553 Japan; 2 Department of Public Oral Health, Program of Oral Health Sciences, Graduate School of Biomedical and Health Sciences, Hiroshima University, Kasumi 1-2-3, Minami-Ward, Hiroshima, 734-8553 Japan

**Keywords:** Anti-angiogenesis, Integrin αvβ_3_, MK-0429, Oral squamous cell carcinoma

## Abstract

**Purpose:**

Integrin αvβ_3_ is an essential molecule for tumor angiogenesis. This study aimed to investigate the anti-tumor effect of MK-0429, an integrin αvβ_3_ antagonist, on oral squamous cell carcinoma (OSCC) through its inhibitory effect on angiogenesis.

**Methods:**

In this study, we investigated the effect of MK-0429 on cellular function and angiogenesis in vitro with the use of an immortalized human umbilical vein endothelial cell, HUEhT-1, which is immortalized by the electroporatic transfection of hTERT. The effect of MK-0429 on the integrin αvβ_3_ signaling pathway was examined by FAK, MEK1/2 and ERK 1/2 phosphorylation. The anti-angiogenic effect of MK-0429 was evaluated by in vitro tube formation assay. The anti-tumor effect on OSCC was assessed by administrating MK-0429 to mouse oral cancer xenografts.

**Results:**

MK-0429 inhibited cell proliferation, migration, and adhesion of HUEhT-1 in a dose-dependent manner. FAK, MEK and ERK phosphorylation were significantly blocked by MK-0429 treatment. Tube formation was suppressed by MK-0429 in dose-dependent manner. Tumor progression was significantly suppressed by MK-0429 administration in mouse oral cancer xenografts. Histological study revealed that MK-0429 decreased tumor vascularization.

**Conclusion:**

These results indicated integrin αvβ_3_ as a therapeutic target for OSCC and suggested that MK-0429 might be clinically applicable as an anti-tumor agent with potent anti-angiogenic activity*.*

**Supplementary Information:**

The online version contains supplementary material available at 10.1007/s00432-022-04100-3.

## Introduction

Integrin αvβ_3_ is homeostatically expressed in vascular endothelial cells and plays an essential role in angiogenesis through its involvement in endothelial cell adhesion and migration (Brooks et al. 1994a, 1994b; Avraamides et al. 2008). Integrin αvβ_3_ is also expressed in osteoclasts, where it promotes bone resorption through adhesion to the extracellular matrix (McHugh et al. 2000). Therefore, integrin αvβ_3_ is essential for tumor progression, and the regulation of integrin αvβ_3_ is expected to inhibit angiogenesis and bone metastasis in cancer. However, successful targeting of integrin αvβ_3_ molecular agents has not led to their clinical application. The clinical trials of intetumumab (a human anti-integrin αvβ_3_ monoclonal antibody) (O’Day et al. 2011), Vitaxin, etaracizumab (a human anti-integrin αv monoclonal antibody) (McNeel et al. 2005; Hersey P, Sosman J, O’Day S, Richards J, Bedikian A, Gonzalez R, Sharfman W, Weber R, Logan T, Buzoianu M, Hammershaimb L, Kirkwood JM, Etaracizumab Melanoma Study G 2010), and cilengitide (a peptidic integrin αvβ_3_/αvβ_5_ inhibitor) (Stupp et al. 2014a; Manegold et al. 2013; Vermorken et al. 2014) have not demonstrated increased beneficial anti-tumor effects for solid tumors, glioblastoma, or malignant melanoma alone or in combination over the standard regimen. On the contrary, low doses of cilengitide have produced unexpected results, such as increased tumor angiogenesis and tumor growth in mouse xenograft models of B16F0 melanoma or Lewis lung carcinoma (Reynolds et al. 2009).

Therefore, we focused on MK-0429, a potent Arg-Gly-Asp (RGD) mimetic integrin αvβ_3_ antagonist, which is a non-peptide small molecule with good oral bioavailability in humans. Hutchinson et al. succeeded in developing MK-0429 using imidazolidinone, a derivative of urea, as a starting material, and when administered to osteoporosis model mice, it restored almost an equal amount of bone mass as in non-osteoporosis mice (Hutchinson et al. 2003). In a mouse melanoma lung metastasis model, MK-0429 administration significantly inhibited the progression of metastases and reduced the tumor volume compared to that of the control group (Pickarski et al. 2015). In a small clinical study of hormone-refractory prostate cancer patients with metastatic bone disease, significant reductions in the bone metastasis marker urinary N-telopeptide were observed without major adverse events (Rosenthal et al. 2010). These emerging evidences suggest that MK-0429 is potent as a novel molecule-targeting therapeutic agent that considers integrin αvβ_3_ as a target molecule.

In this study, using an immortalized human umbilical vein endothelial cell (HUVEC), HUEhT-1, we evaluated the effect of MK-0429 on angiogenesis in vitro. HUEhT-1 is an immortalized HUVEC by electroporation of pIRES-hTERT-hygr. HUEhT-1, as with wild-type HUVEC, has a vascular endothelial cell-like morphology and has been shown to have normal characteristics of vascular endothelial cells, such as vWF expression and tube formation (Anno et al. 2007). In addition, we evaluated the anti-tumor effects of MK-0429 against oral squamous cell carcinoma (OSCC) using human oral cancer xenograft models. MK-0429 is originally an orally administered drug; however, in this study, we administrated MK-0429 to xenografts by a subcutaneous implanted osmotic minipump to maintain an accurate dose.

## Materials and methods

### Cell culture and reagents

HUEhT-1 (JCRB1458) cells were purchased from Japanese Collection of Research Bioresources (JCRB) Cell Bank (Osaka, Japan). They were cultured at 37 °C in a humidified atmosphere in 5% CO_2_ in air and maintained with endothelial cell growth medium supplemented with 0.02 ml/ml fetal calf serum, 5 ng/ml recombinant human epidermal growth factor, 10 ng/ml recombinant human basic fibroblast growth factor, 20 ng/ml insulin-like growth factor, 0.5 ng/ml recombinant human vascular growth factor-165, 1.0 µg/ml ascorbic acid, 22.5 µg/ml heparin, and 0.2 µg/ml hydrocortisone (Promo Cell, Heidelberg, Germany). Culture plates and dishes were coated with 0.5 µg/cm^2^ vitronectin (A14700, Gibco, Carlsbad, CA, USA) in advance of cell culture.

A human tongue squamous cell carcinoma cell line, SAS (JCRB0260) cells, were also purchased from JCRB Cell Bank. The cells were cultured at 37 °C in a humidified atmosphere in 5% CO_2_ in air and maintained with Dulbecco’s modified eagle medium (DMEM; Sigma-Aldrich, St. Louis, MO, USA) supplemented with 10% fetal bovine serum (FBS), 100 IU/mL penicillin, and 100 µg/mL streptomycin (Gibco).

An immortalized human oral keratinocyte cell line, RT7 cells, were established by transfection of hTERT and E7, as previously described (Fujimoto et al. 2004). The cells were cultured at 37 °C in a humidified atmosphere in 5% CO_2_ in air and maintained with KGM-Gold Bullet Kit (Lonza, Switzerland) culture medium.

MK-0429 was purchased from MedChemExpress (Monmouth Junction, NJ, USA). A stock solution of MK-0429 were reconstituted with dimethyl sulfoxide (DMSO) (Sigma-Aldrich). In vitro, the stock solution was diluted with culture medium prior to use.

### Cell proliferation assay

The proliferation of culture cells was evaluated by determining the number of viable cells using Cell Counting Kit-8 (Dojindo Laboratories, Kumamoto, Japan) according to the manufacturer’s instructions. After incubation of cells for 24, 48, or 72 h in 96-well plates with the indicated various concentrations of MK-0429, kit reagent WST-8 was added to the medium and incubated for another 2 h. The absorbance of samples (450 nm) was determined using 800TS™ Absorbance Microplate Reader (BioTek Instruments Inc, Winooski, VT, USA).

### Lactate dehydrogenase assay

The cytotoxicity of MK-0429 was evaluated by measuring the lactate dehydrogenase (LDH) activity using Lactate Dehydrogenase Activity Assay Kit (MAK066, Sigma-Aldrich). After incubation of HUEhT-1 cells for 24 h in 96-well plates with the indicated various concentrations of MK-0429, culture supernatant was harvested. Reagents were mixed to prepare samples following the manufacturer’s protocol. The absorbance of samples (490 nm) was determined using 800TS™ Absorbance Microplate Reader (BioTek Instruments Inc).

### Wound healing assay

The cells were cultured in 12-well plates until reaching a confluent monolayer, when they were scratched with a 200 µl pipette tip. The cells were washed with phosphate buffered saline (PBS), and the indicated amount of MK-0429 was added to the medium and incubated for 24 h. The same section of the wound size of pre- and post-incubation were observed under a phase-contrast microscopy (BZ-9000; Keyence Corporation, Osaka, Japan), and the reduction rate of the wound area was calculated.

### Adhesion assay

After harvesting more than 5.0 × 10^6^ cells of each cell line, the cells were divided into four groups and pre-treated with the intended concentration of MK-0429 for one hour at 37 °C. Each cell suspension was adjusted in serum-free optimal medium for each cell line to 2.0 × 10^5^ cells/500 µl in HUEhT-1 or 1.0 × 10^5^ cells/500 µl in RT7 and SAS. The cells were seeded into 24-well plates precoated with vitronectin and allowed to stand for one hour at 37 °C in a humidified atmosphere in 5% CO_2_ in air. After rinsing the medium with PBS, adherent cells were fixed with 10% neutral buffered formalin solution and stained with methylene blue solution and measured under microscopic observation.

### Western blotting

Following incubation with the culture media, HUEhT-1 cells were cultured in the presence of the indicated concentration of MK-0429 for two days. The cells were harvested, and their protein was extracted using homogenization in a radioimmunoprecipitation assay buffer (Nacalai Tesque, Kyoto, Japan). Western blotting was performed according to our previous study (Nakagawa et al. 2020). In brief, the protein concentration was determined using a Pierce™ BCA Protein Assay kit (Thermo Fisher Scientific, Waltham, MA, USA). From each sample, 20 µg of protein was electrophoresed in 5–20% sodium dodecyl sulfate–polyacrylamide electrophoresis gradient gels (E-T520L, ATTO Corp, Tokyo, Japan) and transferred onto a polyvinylidene difluoride membrane. Non-specific binding was blocked in Tris-buffered saline (TBS) containing a chemical blocking reagent (Ez Block Chemi, ATTO Corp.) and 0.1% Tween-20 for one hour at room temperature. The membranes were incubated with the following primary antibodies: integrin alpha V rabbit mAb (ab179475; at 1: 1000), FAK rabbit mAb (ab40794; at 1: 1000), p-FAK (Y397) rabbit mAb (ab81298; at 1: 1000), (Abcam Inc., Cambridge, MA, USA), MEK 1/2 rabbit pAb (#9122; at 1: 1000), p-MEK 1/2 rabbit mAb (#9154; at 1: 1000), Erk 1/2 rabbit mAb (137F5; at 1: 1000), p-Erk 1/2 rabbit mAb (20G11; at 1: 1000) (Cell Signaling Technology, Danvers, MA, USA), and GAPDH mouse mAb (#MAB374 at 1:2000; Millipore, Billerica, MA, USA) at 4 °C overnight. Following washing with TBS-T, the membranes were incubated with a horseradish peroxidase-conjugated secondary antibody (GE Healthcare Bio-Sciences) diluted in TBS-T with chemical blocking reagent described above for one hour at room temperature. The proteins of interest were then visualized using an ECL Advance Western Blotting Detection kit (GE Healthcare Bio-Sciences) on the LAS 4000 Mini-Imaging system (Fujifilm, Tokyo, Japan).

### Tube formation assay

Tube formation assay was performed using Angiogenesis Assay Kit (Promo Cell) according to the manufacturer’s protocol. Extracellular Matrix Solution (Promo Cell) was applied to the EZVIEW Glass Bottom Assay Plate 96 well (AGC Techno Glass, Shizuoka, Japan). After harvesting more than 9.0 × 10^5^ HUEhT-1 cells, the cells were resuspended in serum-free endothelial cell growth medium (Promo Cell) with 100 ng/ml VEGF_165_ (Peprotech, Rocky Hill, NJ, USA). After adding MK-0429 to achieve the indicated various concentrations, 2.0 × 10^4^ cells/well were seeded. As an inhibitor control, suramin was administered to the reference cell at a final concentration of 10 µM. The plate was incubated at 37 °C, 5% CO_2_, and 95% humidity for 16 h. After the cells were washed, fluorescent staining dye was applied to each well, and each cell was examined with a fluorescent phase-contrast microscope (BZ-9000; Keyence Corporation) by bright-field and fluorescent (FTIC/eGFP) field. Tube formation was measured on the images using Image J software (Angiogenesis Analyzer for Image J; US National Institutes of Health, Bethesda, MD, USA). The number of junctions, number of meshes, number of segments, and total length of segments were calculated for each image.

### Animal experiments

The animal experimental protocol was reviewed and approved by Review Board of Animal Experiment Committee of Hiroshima University (approval no. A20-158). Four-weeks-old female NOD/SCID mice (CLEA Japan, Inc. Tokyo, Japan) were housed in a temperature and humidity-controlled facility under 12 h of light: 12 h of dark cycle. Animals had ad libitum access to food and water. A xenograft model of human oral cancer was established by inoculating 1.0 × 10^7^ cells of SAS subcutaneously in the posterior neck. MK-0429 was administrated by osmotic minipump. MK-0429 was formulated in 50% DMSO/50% distilled water at a concentration of 20 mg/mL. MK-0429 or a vehicle solution were filled in minipumps (Alzet, # 1004, flow rate 0.11 μL/h). Minipumps were placed in mice subcutaneously in a pocket on the back. The total amount of MK-0429 administered by osmotic pump for 28 days was calculated to be 100 mg/kg. The mice were killed 28 days after tumor inoculation, and tumors were extracted. Tumor weight was measured by a digital scale (Sartorius Entris 5201-1S; Göttingen, Germany), and tumor volume was calculated from the Eq. 4π/3 × (*R*_1_ / 2 + *R*_2_ / 2)^3^, where *R*_1_ = longitudinal radius, and *R*_2_ = transverse radius measured by a caliper.

### Histological examinations

Tumor specimens of mouse OSCC xenografts were fixed in 10% buffered formalin and dehydrated in a graded alcohol series. Specimens were then embedded in paraffin and cut into 4 µm thick sections using a microtome. The sections were de-paraffinized with xylene and rehydrated in graded alcohols and stained with hematoxylin and eosin (HE) according to standard protocols. Immunohistochemistry (IHC) was performed with primary antibodies against Vascular Endothelial Growth Factor Receptor 2 (VEGFR2) (rabbit mAb, 55B11; at 1:1000; Cell Signaling Technology), integrin alpha V rabbit mAb (ab179475; at 1: 500), CD31 rabbit mAb (ab76533; at 1: 500) (Abcam Inc.), α-Smooth Muscle Actin (αSMA) (rabbit mAb, ARG66381; at 1: 2000), Ki-67 (rabbit mAb, ARG66347; at 1: 200)(Arigo Biolaboratories Corp, Hsinchu City, Republic of China). The sections were incubated with the primary antibodies at 4 °C for 12 h and visualized with phase-contrast microscopy (BZ-9000; Keyence Corporation).

### Statistical analysis

All experiments were repeated at least three times throughout the study. Statistical analysis was performed using Student’s *t* test using SPSS version 23.0 (IBM Corp., Armonk, NY, USA). Results were described as the mean ± SD. Differences were considered statistically significant at *P* < 0.05.

## Results

### Inhibitory effect of MK-0429 on HUEhT-1 cell growth, migration, and adhesion

**Fig. 1:**
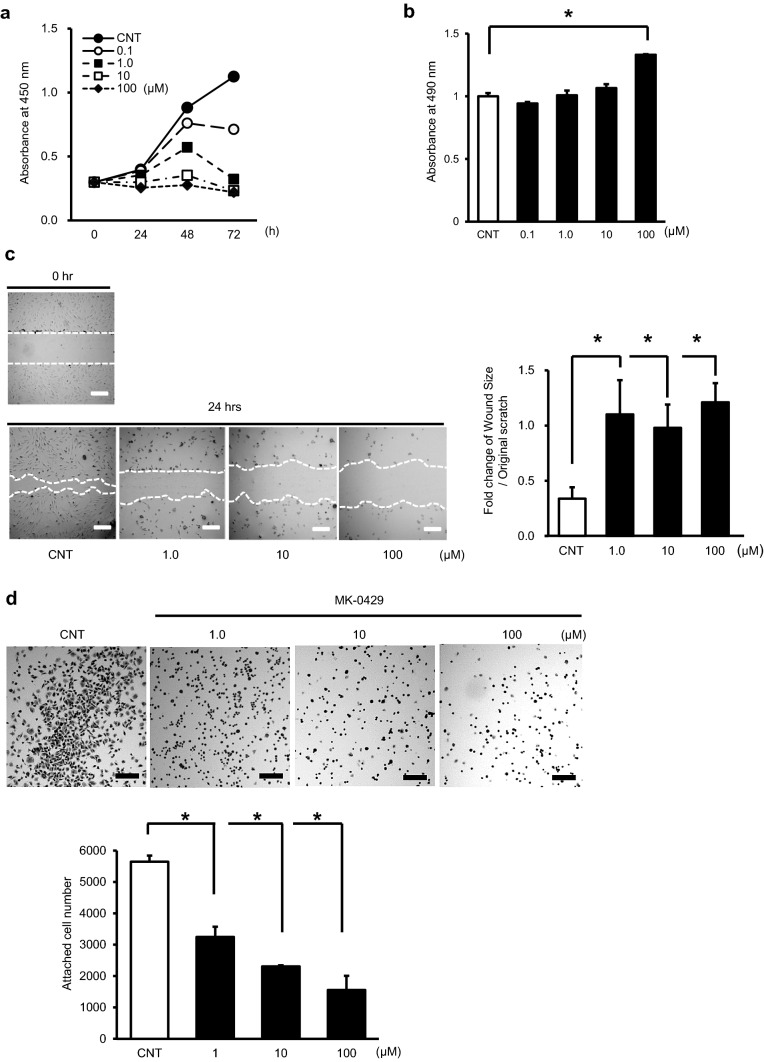
Effects of MK-0429 on immortalized human umbilical vein endothelial cell, HUEhT-1. **a** Effect of MK-0429 on the growth of HUEhT-1 cells. HUEhT-1 cells were treated in the presence of the indicated amount of MK-0429 or dimethyl sulfoxide (DMSO) as a control for 24, 48, 72 h, and CCK-8 assay was performed. **b** The cytotoxicity of MK-0429 against HUEhT-1 cells. HUEhT-1 cells were incubated with the indicated concentration of MK-0429 or DMSO for 24 h, and the amount of lactate dehydrogenase (LDH) in the culture medium was measured. **c** Cell migration assay of HUEhT-1 cells under MK-0429 treatment. Representative figures at each concentration of MK-0429 are shown in panels. A white bar indicates 200 µm. The graph shows the reduction rate for each concentration of MK-0429 relative to the original scratch area. **d** Adhesion assay of HUEhT-1 cells onto substrates coated with vitronectin under MK-0429 pretreatment. The graph shows the number of attached cells. A black bar indicates 200 µm. Each experiment was performed more than 5 times and obtained similar results. Values are presented as the mean ± standard error of mean (*P < 0.05)

To determine the effects of MK-0429 on various cellular functions of HUEhT-1, we performed cell proliferation assay, cytotoxicity assay, migration assay, and cell adhesion assay. The proliferation of HUEhT-1 was inhibited in a dose-dependent manner by MK-0429 (Fig. 1a). The cytotoxicity of MK-0429 was assessed by measuring the LDH in culture medium. At high concentrations of MK-0429, the amount of LDH in culture medium increased; however, there was no dose-dependent increase (Fig. 1b). Next, we performed wound healing assay to evaluate the effect of MK-0429 on cell migration. Cell migration was independent of the concentration and was also suppressed by a small dose (1.0 µM) of MK-0429 (Fig. 1c). We also evaluated the effect of MK-0429 on cell adhesion to vitronectin, an extracellular matrix, which was applied to culture plates. The results showed that MK-0429 dose-dependently decreased the cell adhesion ability of HUEhT-1.

### MK-0429 inhibited integrin αvβ_3_ activation

**Fig. 2:**
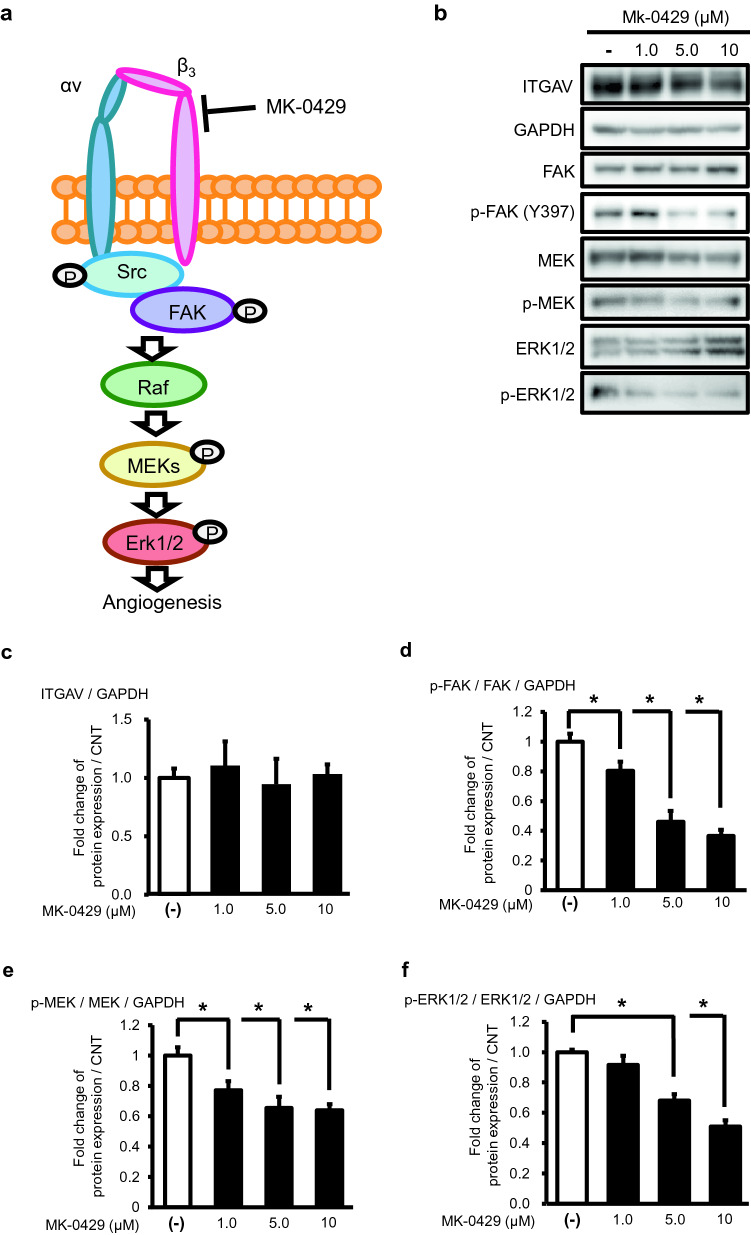
Evaluation of MK-0429-induced activation of integrin signaling. **a** Schematic diagram of integrin αvβ_3_-FAK-MEK1/2-ERK1/2 pathway and inhibition by MK-0429. Integrin αv and β_3_ assembly activates Raf-MEK-ERK pathway dependent on phosphorylation of FAK phosphorylation thereby promote tumor vascularization. MK-0429 mimics RGD peptides and inactivates the integrin αvβ_3_ signaling pathway. **b **Panels of western blotting of integrin αv (ITGAV), FAK, phospho-FAK (Y397), MEK1/2, phospho-MEK1/2, ERK1/2, phospho-ERK1/2, and glyceraldehyde-3-phosphate dehydrogenase (GAPDH) after treatment with the indicated concentration of MK-0429. **c** The expression status of ITGAV was evaluated quantitatively as a fold change compared with the internal control, GAPDH. **d**–**f** Phosphorylation of FAK, MEK1/2 and ERK1/2 were evaluated quantitatively as a fold change compared with the total FAK, MEK1/2 and ERK 1/2 that corrected with the concentration of GAPDH band. Each experiment was performed more than 5 times and similar results were obtained. Values were presented as the mean ± standard error of mean (*P < 0.05)

Assembly of integrin αv and β_3_ promotes tumor vascularization through FAK phosphorylation and activation of the MEK-ERK pathway (Avraamides et al. 2008). MK-0429, an RGD peptide mimetic antagonist, is considered to inhibit angiogenesis by suppressing this integrin-FAK signaling pathway through blocking integrin assembly (Fig. 2a). To determine whether the various effects of MK-0429 on HUEhT-1 cells were due to the suppression of integrin αvβ_3_ activation, we examined the effects of MK-0429 on integrin αvβ_3_ expression and the signaling pathway. As a result of quantification by the ratio of the expression of endogenous GAPDH, the expression of integrin αvβ_3_ was not changed by MK-0429 treatment (Fig. 2b, c). On the other hand, phosphorylation of FAK, MEK1/2 and ERK 1/2 was reduced by MK-0429 treatment, whereas the total expression levels of FAK, MEK1/2 and ERK 1/2 were stable (Fig. 2b). Quantification of expression levels by the ratio of total to phosphorylation that were corrected by the expression of endogenous GAPDH showed statistically significantly decreased phosphorylation of FAK, MEK1/2 and ERK 1/2 (Fig. 2d, f). These results indicated that MK-0429 inhibits integrin αvβ_3_ activation and suppresses cell function.

### MK-0429 suppressed in vitro angiogenesis in HUEhT-1 cells

**Fig. 3:**
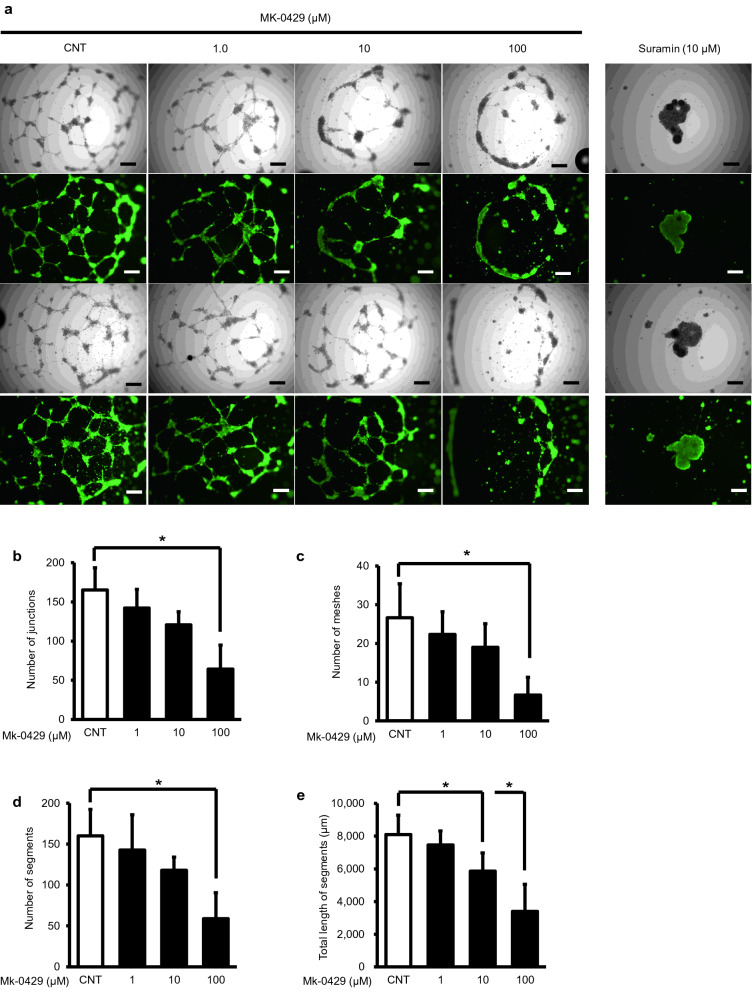
Evaluation of the inhibitory effect of MK-0429 on angiogenesis in vitro. **a** Panels of phase-contrast and fluorescent images tube formation assay. Images of two representative locations at various MK-0429 concentration were shown as phase-contrast images (upper) and fluorescence images (lower). Cells treated with suramin (10 μM) were the negative control. Bars in the panels indicated 500 µm. **b**–**e** Quantification of the number of junctions (**b**), number of meshes (**c**), number of segments (**d**), and total length of segments (**e**) by Angiogenesis Analyzer for Image J. Each item was evaluated by measuring five different fields of view. Values were presented as the mean ± standard error of mean of 5 fields (*P < 0.05)

When cultured on an extracellular matrix gel in the presence of angiogenic factors, since HUEhT-1 is derived from HUVECs, the cells adhere to each other to form a luminal structure. To assess the effect of MK-0429 on angiogenesis, we performed a tube formation assay using VEGF_165_ as an angiogenic factor. As shown in (Fig. 3a), while the control group without MK-0429 showed remarkable formation of vascular structures, the MK-0429-treated group showed significantly impaired vascular formation in a dose-dependent fashion. To evaluate objectively the inhibitory effect of MK-0429 on vascular formation, the number of junctions, number of meshes, number of segments, and total length of segments were quantified using image analysis software (Angiogenesis Analyzer for Image J). All outcomes showed a dose-dependent decrease in MK-0429, and the 100 µM group showed a statistically significant inhibition of vascular formation compared to that of the control group (Fig. 3b–e).

### Inhibitory effect of MK-0429 on tumor progression in mouse OSCC xenografts

**Fig. 4:**
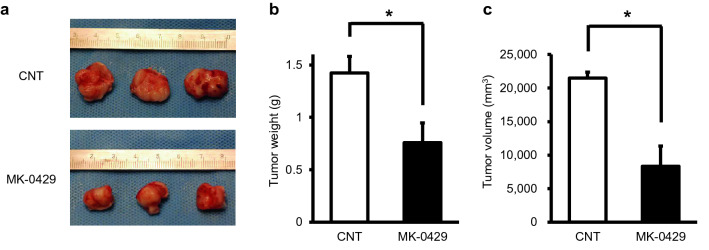
Effect of MK-0429 on mouse OSCC xenograft model. **a** Representative photos of the tumors of mouse OSCC xenografts. Tumors of the control group (DMSO) are shown in the upper panel, and that of the MK-0429-administrated group (100 mg/kg) are shown in the lower panel. **b**, **c** Xenograft tumor weight (**b**) and volume (**c**) after being treated with MK-0429 or DMSO. Values are presented as the mean ± standard error of mean (*N* = 5, *P < 0.05)

To confirm the inhibitory effect of MK-0429 on oral squamous cell carcinoma cells in vitro, we performed each assay of cell proliferation, migration, and adhesion by using the OSCC cell line, SAS. To confirm whether MK-0429 has a specific effect on squamous cell carcinoma, RT7, an immortalized oral squamous cell line, was used as a control. In the CCK-8 assay, the proliferation of SAS was slightly inhibited by MK-0429, whereas that of RT7 was not affected (Supplementary Fig. 1a). However, the migration and adhesion of cells were inhibited in a dose-dependent manner by MK-0429 in both RT7 and SAS with no significant difference between them. Therefore, the MK-0429 effect was not specific to OSCC in vitro (Supplementary Fig. 1b, c). Next, we established mouse oral cancer xenograft models. The SAS cells were injected subcutaneously at the posterior neck of 4-week-old female NOD/SCID mice. MK-0429 was administrated by an osmotic minipump to maintain an accurate administration amount and administration speed. At 28 days after tumor inoculation, xenografts were sacrificed, and tumors were extracted. Tumor growth was markedly inhibited in the MK-0429 group compared to that of the control group (Fig. 4a). The weight and volume of the extracted tumors were reduced with statistical significance in the MK-0429 group (Fig. 4b, c).

### Suppression of tumor vascularization by MK-0429

**Fig. 5:**
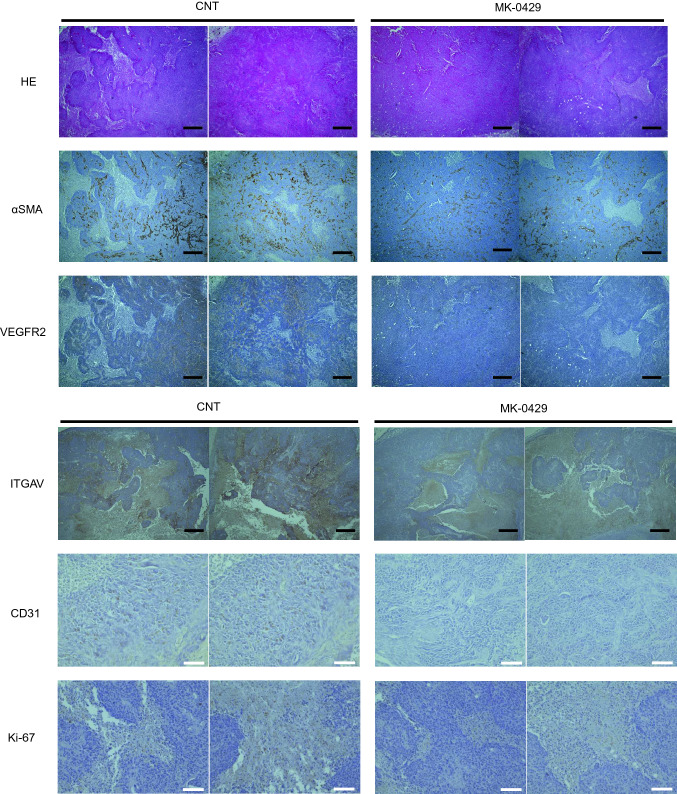
IHC study of tumor specimens of mouse OSCC xenografts. Representative microscopic images of the specimen of xenografts. HE staining and IHC staining for integrin αv, VEGFR2, CD31 (the vascular endothelial cell marker), αSMA (the vascular smooth muscle marker), Ki-67 (cell proliferation marker) in the tumors from the control group (CNT) and MK-0429 treated group (MK-0429). Black bars in HE, integrin αv, VEGFR2 and αSMA panels indicated 500 µm, and white bars in CD31 and Ki-67 panels indicated 100 µm

To investigate tumor vascularization of the mouse OSCC xenograft, we performed IHC of integrin αv, VEGFR2, CD31 (vascular endothelial cell marker), αSMA (vascular smooth cell marker) and Ki-67 (cell proliferation marker) as shown in (Fig. 5). The IHC-stained area of the MK-0429 group was less than that of the control group for both integrin αv, VEGFR2 and αSMA. Moreover, CD31- and Ki-67-positive cells were significantly decreased in MK-0429 group compared with the control group. These results suggested that the inhibitory effect on tumor progression was mainly due to the inhibition of tumor vascularization. Moreover, VEGF-dependent angiogenesis, which has been proven to be compensatively enhanced by integrin inhibitors (Reynolds et al. 2009), was not enhanced under MK-0429 administration.

## Discussion

In the present study, we revealed that MK-0429 has an inhibitory effect on the cellular function of vascular endothelial cells, and these effects were mediated by inhibiting the integrin signaling pathway without interfering with the integrin expression levels. We also elucidated that MK-0429 inhibited angiogenesis in vitro. In addition, MK-0429 showed an inhibitory effect on tumor progression via the suppression of tumor vascularization in mouse oral cancer xenografts. Considering these results, MK-0429 can be developed as a potent anti-tumor agent targeting the integrin αvβ_3_ pathway against OSCC.

The angiogenic effect of integrin αvβ_3_ was reported in 1994 (Brooks et al. 1994a), and its anti-tumor effect was reported as a follow-up report (Brooks et al. 1994b), which attracted a great deal of attention. Since then, various agents have been developed in anticipation of cancer control by integrin inhibition; however, they have not been successful for more than 30 years. The clinical trials in patients with squamous cell carcinoma of the head and neck (SCCHN) have also not demonstrated the efficacy of cilengitide. In the phase II clinical trial of cilengitide combined with cisplatin, 5-fluorouracil, and cetuximab (PFE) versus PFE alone in patients with recurrent and/or metastatic SCCHN (R/M-SCCHN), cilengitide did not improve clinical outcomes (Vermorken et al. 2014). Since that report, there have been a few studies of cilengitide in OSCC; however, they were in vitro studies and did not show drastic efficacy that would lead to clinical application (Heiduschka et al. 2014; Wichmann et al. 2017; Zhang et al. 2019).

In 2007, Reynolds et al. reported an important study on the reasons for the poor clinical outcomes of RGD-mimetic avb_3_ and avb_5_ inhibitors, including cilengitide (Reynolds et al. 2009). They demonstrated in vivo that low concentrations of RGD-mimetic αvβ_3_ and αvβ_5_ inhibitors (cilengitide and S36578) paradoxically promote tumor growth and tumor angiogenesis by promoting VEGF-mediated angiogenesis. These results suggest that the promoting-angiogenic effects of low concentrations of RGD-mimetic integrin inhibitors may compromise their efficacy as anticancer agents.

Cilengitide is a peptide-like RGD, and its absorption, pharmacokinetics, and metabolism in vivo may be similar to those of RGD. The elimination half-life of cilengitide was reported to be 2–4 h, independent of dose (Hariharan et al. 2007). In a phase III study of MGMT methylated glioblastoma (CENTRIC EORTC 26,071–22,072 study), the short half-life of cilengitide was considered to be one of the reasons for failure, suggesting that it may have had an insufficient inhibitory effect on angiogenesis (Stupp et al. 2014a, 2014b; Chinot 2014; Tucci et al. 2014). Therefore, it is expected that continuous administration is necessary to achieve a sufficient therapeutic effect. Although the half-life of MK-0429 was reported to be 3.5 h, which was similar to that of cilengitide (Hutchinson et al. 2003), MK-0429 was designed as orally administration agents (Hutchinson et al. 2003; Pickarski et al. 2015). Oral medications are the preferred dosage form for patient compliance and, consequently, it is easy to maintain the optimal dose continuously. In the present study, as shown in (Fig. 4), continuous administration of MK-0429 significantly suppressed tumor progression. Therefore, it can be expected to have a better therapeutic effect than cilengitide by constructing a dosing schedule that considers pharmacokinetics as an oral administration agent.

Similar to MK-0429, S36578 consists of non-peptide RGD-mimetic small molecules (Perron-Sierra et al. 2002; Maubant et al. 2006). It has been shown that inhibition of angiogenesis by integrin αvβ_3_ inhibitors is mediated by the induction of apoptosis of vascular endothelial cells (Brooks et al. 1994a). S36578 was highly selective for αvβ_3_ and αvβ_5_ integrins and induces detachment, activation of caspase-8, and apoptosis in HUVECs cultured on vitronectin (Perron-Sierra et al. 2002). Apoptosis by S36578 was induced only on the extra cellular matrix (ECM), which served as a ligand for integrins such as vitronectin, but not on interstitial matrices such as fibronectin. The apoptosis induced by s36578 was considered to be anoikis as the result of the loss of integrin-dependent adhesion between ECM and cells. In the present study, the cytotoxicity of MK-0429 to endothelial cells was not significant, as shown in (Fig. 1b); however, the loss of cell adhesion and migration on the vitronectin coating plates by MK-0429 was significant, as shown in (Fig. 1c, d). These results indicated that not only S36578 but also MK-0429 inhibit integrin-dependent adhesion to ECM.

Reynolds’ study revealed that low-dose S36578 (0.1 mg/ml) promoted tumor angiogenesis and progression (Reynolds et al. 2009). Moreover, the anti-tumor effects of B16F0 melanoma and the LLC tumor were poor even with high-dose S36578 administration (200 mg/kg by intraperitoneal injection or 100 mg/ml continuous administration by osmotic pump). On the other hand, MK-0429 inhibited angiogenesis in a dose-dependent manner in the VEGF-induced tube formation assay and did not promote VEGF-mediated angiogenesis (Fig. 3). We also demonstrated the optimal amount (100 mg/kg) of MK-0429, which was indicated in the study of osteoporosis (Hutchinson et al. 2003) and B16F10 melanoma (Pickarski et al. 2015) showing marked tumor suppression (Fig. 4). Furthermore, IHC staining indicated that tumor vascularization induced by VEGF–VEGFR2 signaling was remarkably suppressed by MK-0429 (Fig. 5). These results are contrary to the conclusion of Reynolds’ study that RGD-mimetic agents contribute to tumor growth and angiogenesis via VEGF-mediated angiogenesis. Although MK-0429 and s36578 are similar agents in terms of non-peptide RGD-mimetic small molecules, MK-0429 is superior to s36578 in that the therapeutic effect was obtained in vivo at an optimal dose.

Although several reports showed anti-tumor effects of MK-0429 in preclinical studies (Pickarski et al. 2015; Rosenthal et al. 2010), no clinical trials have been conducted to date. Therefore, it was supposed that MK-0429 had not demonstrated sufficient therapeutic efficacy for clinical application. There are also a lack of reports of preclinical studies that provide a basis for proceeding to clinical trials. The present study was the first report to investigate MK-0429 as an angiogenesis inhibitor, yet there have been no reports of MK-0429 as an angiogenesis inhibitor in either preclinical studies or clinical trials. In the future, MK-0429 may be combined with existing therapies in carcinomas that have already been demonstrated to respond to angiogenesis inhibitors.

In conclusion, we demonstrated that MK-0429 had not indicated a remarkable anti-tumor effect to OSCC in vitro, and the progression of tumors disseminated to mouse OSCC xenografts was inhibited by MK-0429 administration by suppressing tumor vascularization. Therefore, existing integrin αvβ_3_-targeting agents including MK-0429 should be re-examined for their anti-tumor effects as angiogenesis inhibitors. Further studies are expected for the development of novel integrin αvβ_3_ inhibitors and its application to clinical trials.

## Supplementary Information

Below is the link to the electronic supplementary material.

Supplementary file1 Effects of MK-0429 on immortalized human oral keratinocyte, RT7, and oral squamous cell line, SAS. (a) Effect of MK-0429 on the growth of RT7 and SAS. The cells were treated in the presence of the indicated amount of MK-0429 or dimethyl sulfoxide (DMSO) as a control for 24, 48, and 72 hours, and CCK-8 assay was performed. (b) Cell migration assay of RT7 and SAS under MK-0429 treatment. Representative images of cells with or without MK-0429 treatment (10 µM) are shown. A white bar indicated 200 µm. The graph showed the reduction rate for each concentration of MK-0429 relative to the original scratch area. (c) Adhesion assay of RT7 and SAS onto substrates coated with vitronectin under MK-0429 pretreatment. The graph showed the number of attached cells. A black bar indicated 200 µm. Each experiment was performed three times and obtained similar results. Values are presented as the mean ± standard error of mean (*P < 0.05) (PPTX 1923 KB)
