# Supplementary material for: Inhibition of angiogenesis and tumor progression of MK-0429, an integrin αvβ3 antagonist, on oral squamous cell carcinoma
Source: J Cancer Res Clin Oncol. 2022 Jun 17;148(12):3281–92. doi: 10.1007/s00432-022-04100-3 (PMC9587112; doi:10.1007/s00432-022-04100-3)
Supplement: Supplementary file 1 — Supplementary file1 Effects of MK-0429 on immortalized human oral keratinocyte, RT7, and oral squamous cell line, SAS. (a) Effect of MK-0429 on the growth of RT7 and SAS. The cells were treated in the presence of the indicated amount of MK-0429 or dimethyl sulfoxide (DMSO) as a control for 24, 48, and 72 hours, and CCK-8 assay was performed. (b) Cell migration assay of RT7 and SAS under MK-0429 treatment. Representative images of cells with or without MK-0429 treatment (10 µM) are shown. A white bar indicated 200 µm. The graph showed the reduction rate for each concentration of MK-0429 relative to the original scratch area. (c) Adhesion assay of RT7 and SAS onto substrates coated with vitronectin under MK-0429 pretreatment. The graph showed the number of attached cells. A black bar indicated 200 µm. Each experiment was performed three times and obtained similar results. Values are presented as the mean ± standard error of mean (*P < 0.05) (PPTX 1923 KB) [file 432_2022_4100_MOESM1_ESM.pptx]

## Slide 1
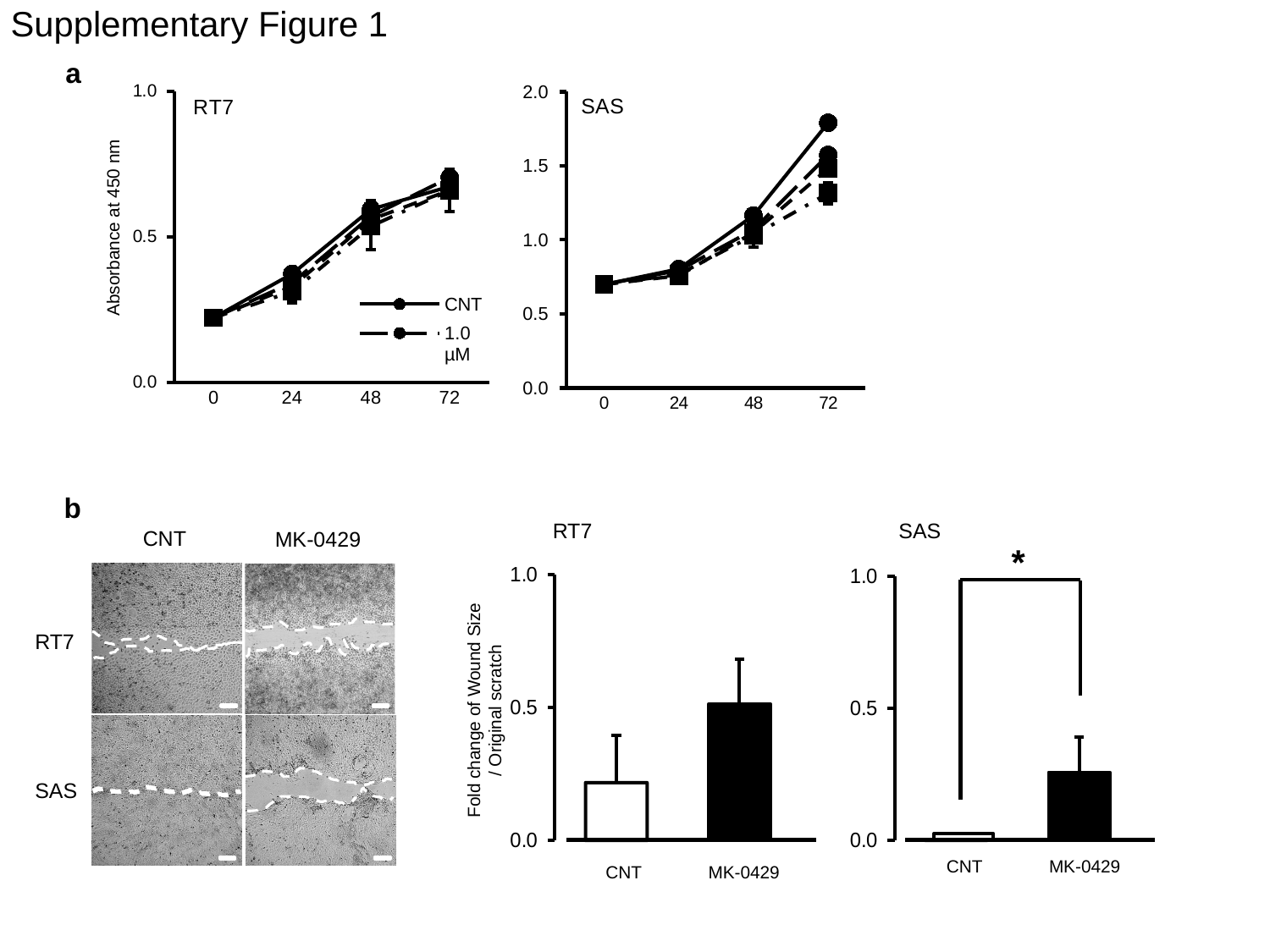

Supplementary Figure 1
a
### Chart: SAS
| Category | CNT | 1.0 µM | 10 µM | 100 µM |
|---|---|---|---|---|
| 0 | 0.699 | 0.699 | 0.699 | 0.699 |
| 24 | 0.8033333333333333 | 0.7936666666666667 | 0.758 | 0.767 |
| 48 | 1.1643333333333332 | 1.068 | 1.0493333333333332 | 1.0303333333333333 |
| 72 | 1.790333333333333 | 1.5730000000000002 | 1.4823333333333333 | 1.3143333333333334 |
### Chart: RT7
| Category | CNT | 1.0 µM | 10 µM | 100 µM |
|---|---|---|---|---|
| 0 | 0.22166666666666668 | 0.22166666666666668 | 0.22166666666666668 | 0.22166666666666668 |
| 24 | 0.37266666666666665 | 0.342 | 0.329 | 0.312 |
| 48 | 0.594 | 0.5703333333333334 | 0.5589999999999999 | 0.536 |
| 72 | 0.6723333333333333 | 0.703 | 0.6593333333333334 | 0.6583333333333333 |Absorbance at 450 nm
b
RT7
SAS
CNT
MK-0429
*
### Chart
| Category | |
|---|---|
| CNT | 0.21733479646141532 |
| MK-0429 | 0.5133699931715298 |
### Chart
| Category | |
|---|---|
| CNT | 0.0 |
| MK-0429 | 0.2574345959755046 |RT7
Fold change of Wound Size
/ Original scratch
SAS
CNT
MK-0429
CNT
MK-0429

## Slide 2
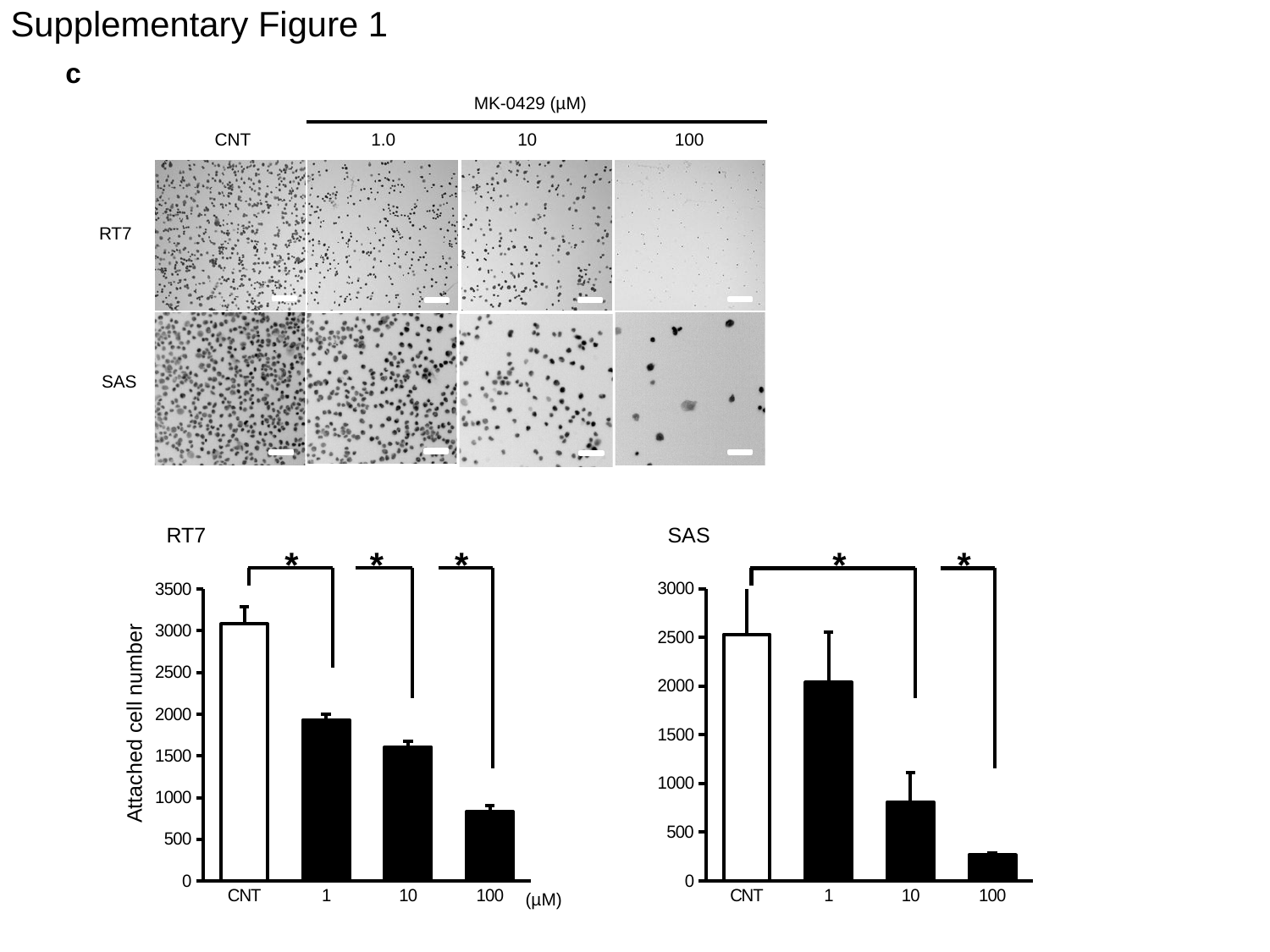

Supplementary Figure 1
c
MK-0429 (µM)
CNT
1.0
10
100
RT7
SAS
RT7
SAS
*
*
*
*
*
### Chart
| Category | |
|---|---|
| CNT | 2531.6666666666665 |
| 1 | 2038.0 |
| 10 | 810.0 |
| 100 | 269.3333333333333 |
### Chart
| Category | |
|---|---|
| CNT | 3080.3333333333335 |
| 1 | 1931.6666666666667 |
| 10 | 1606.3333333333333 |
| 100 | 833.6666666666666 |Attached cell number
 (µM)
